# Supplementary material for: Examining the Drivers of Racial/Ethnic Disparities in Non-Adherence to Antihypertensive Medications and Mortality Due to Heart Disease and Stroke: A County-Level Analysis
Source: Int J Environ Res Public Health. 2021 Dec 2;18(23):12702. doi: 10.3390/ijerph182312702 (PMC8657217; doi:10.3390/ijerph182312702)
Supplement: Supplementary file 1 [file ijerph-18-12702-s001.zip › ijerph-1461750-supplementary.pdf]

Supplemental results

Table S1. Distribution of the features of each construct of determinants of health, overall and by geographical regions.

Variable	Weight	All	Midwest	Northeast	South	West
**Health Behaviors**						
Adult smoking (%)	0.1	18.6	18.5	16	19.4	13.8
Adult obesity (%)	0.05	31.2	30.8	26.5	32.4	24.9
Excessive drinking (%)	0.03	16.1	18.7	18.2	15.2	18.3
Alcohol driving death (%)	0.03	30.8	32.4	28.3	30.8	31.8
Sexually transmitted infections (# per 100,000)	0.03	458.4	461.5	351.9	476.2	402.5
Teen births (# per 1,000)	0.03	43.5	35.4	22.8	48.3	34.1
Food environment index	0.02	6.8	7.1	7.9	6.6	7.4
Physical inactivity (%)	0.02	27.4	25.1	23.6	29	17.9
Access to exercise (%)	0.01	68.4	81.6	87.6	62.2	87.1
**Clinical Care**						
Uninsured (%)	0.05	17.7	12.9	11.1	19.3	18.3
Preventable hospital stays (# per 1,000)	0.05	62.2	60.6	56.6	64.9	40.3
Primary care physicians ratio	0.03	2327.8	1577.9	1473.8	2629.3	1493.8
Diabetic monitoring (%)	0.03	85	86.2	86.1	84.8	82
Mammography screening (%)	0.03	61.9	64.2	63.6	61.5	59.6
Dentist ratio	0.01	3040.8	1986.1	1576.2	3516.7	1640.7
Mental health provider ratio	0.01	2207.5	765	694.5	2781.7	453.5
**Social Economic Factors**						
Unemployment (%)	0.1	6.8	6.2	6.3	6.9	7.8
Children in poverty (%)	0.08	25.4	21.7	18.3	27.3	21.5
High school graduation (%)	0.05	82.1	84.1	84.3	81.5	82.4
Some college education (%)	0.05	56.5	64.7	64.7	53.6	61.2
Income ratio	0.03	4.8	4.5	4.8	4.8	4.7
Single parents households (%)	0.03	37.4	35.1	32.4	38.8	31.8
Social association rate (# per 10,000)	0.03	11.5	12	10.4	11.9	6.7
Violent crime rate (# per 100,000)	0.03	373	378.4	319.5	376.8	402.4
Injury death (# per 100,000)	0.03	69.3	61.7	53	73.4	59.6
**Physical Environment (PE)**						
Air pollution (PM2.5)	0.03	11.8	12.6	11.6	11.9	9.2
Severe housing problems (%)	0.02	16.5	15	18.6	16	23.7
Driving alone (%)	0.02	80.9	82.6	75	81.8	74.2
Long commuting driving alone (%)	0.01	33.3	26.9	35.4	34.2	31.2

Table S2. Distribution of prevalence of AHM non-adherence measures by geographical regions.

	Non-adherence prevalence (%)				
	**All**	**Midwest**	**Northeast**	**South**	**West**
Overall	27.18 (26.93; 27.44)	22.48 (22.04; 22.92)	22.62 (22.03; 23.20)	28.68 (28.43; 28.92)	25.82 (24.83; 26.81)
White	25.41 (25.20; 5.63)	21.58 (21.19; 21.98)	21.46 (21.04; 21.89)	26.68 (26.48; 26.89)	24.03 (23.36; 24.69)
Black	34.50 (34.29; 34.72)	32.21 (31.83; 32.59)	31.90 (31.45; 32.36)	35.43 (35.18; 35.67)	31.95 (31.06; 31.85)
Racial disparity measure					
Prevalence rate ratio (PRR)	1.37 (1.36; 1.37)	1.50 (1.48; 1.53)	1.49 (1.47; 1.52)	1.33 (1.33; 1.34)	1.33 (1.30; 1.37)

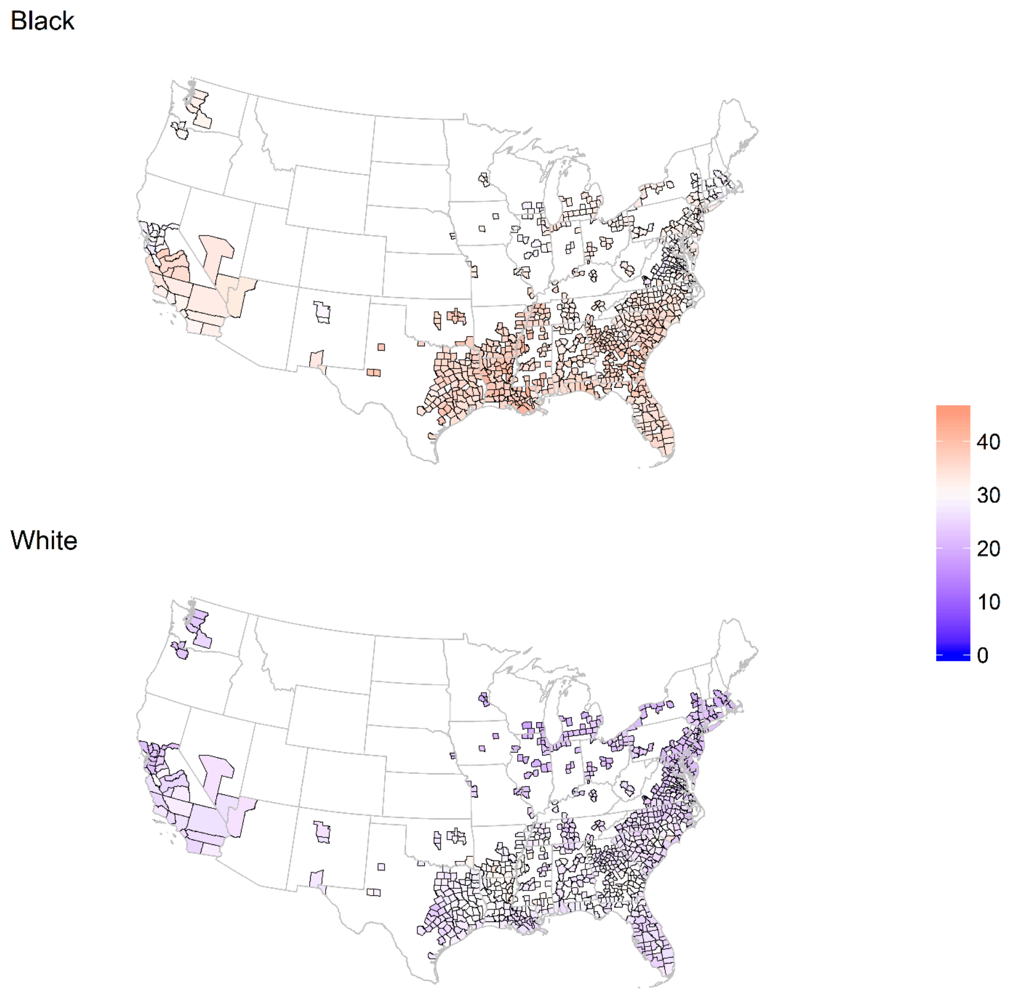

Figure S1. Spatial distribution of AHM non-adherence prevalence for BAAs and nHWs among 875 counties in the US.
